# Combinational Proanthocyanidins and Resveratrol Synergistically Inhibit Human Breast Cancer Cells and Impact Epigenetic–Mediating Machinery

**DOI:** 10.3390/ijms19082204

**Published:** 2018-07-27

**Authors:** Yifeng Gao, Trygve O. Tollefsbol

**Affiliations:** 1 Department of Biology, University of Alabama at Birmingham, 1300 University Boulevard, Birmingham, AL 35294, USA; mvgold@uab.edu; 2 Comprehensive Center for Healthy Aging, University of Alabama Birmingham, 1530 3rd Avenue South, Birmingham, AL 35294, USA; 3 Comprehensive Cancer Center, University of Alabama Birmingham, 1802 6th Avenue South, Birmingham, AL 35294, USA; 4 Nutrition Obesity Research Center, University of Alabama Birmingham, 1675 University Boulevard, Birmingham, AL 35294, USA; 5 Comprehensive Diabetes Center, University of Alabama Birmingham, 1825 University Boulevard, Birmingham, AL 35294, USA

**Keywords:** grape seed proanthocyanidins, resveratrol, breast cancer, synergism, epigenetics

## Abstract

Breast cancer is the second most common cancer and the second leading cause of death from cancer among women in the United States (US). Cancer prevention and therapy through the use of phytochemicals that have epigenetic properties has gained considerable interest during the past few decades. Such dietary components include, but are not limited to, grape seed proanthocyanidins (GSPs) and resveratrol (Res), both of which are present in red wine. In this study, we report for the first time the synergistic effects of GSPs and Res on inhibiting MDA-MB-231 and MCF-7 human breast cancer cells. Our results of 3-(4,5-dimethylthiazol-2-yl)-2,5-diphenyltetrazolium bromide (MTT) assays and clonogenic assays indicate that treatments with the combinations of GSPs and Res synergistically decreased cell viability and posttreatment cell proliferation in both cell lines. Additional analyses show that treatments with GSPs and Res in combination synergistically induced apoptosis in MDA-MB-231 cells by upregulating Bax expression and down-regulating Bcl-2 expression. DNA methyltransferase (DNMT) activity and histone deacetylase (HDAC) activity were greatly reduced in MDA-MB-231 and MCF-7 cells after treatments with GSPs and Res in combination. Collectively, our findings suggest that GSPs and Res synergistically inhibit human breast cancer cells through inducing apoptosis, as well as modulating DNA methylation and histone modifications.

## 1. Introduction

Breast cancer is the second most common cancer and the most common invasive cancer among women in the United States (US). Preceded only by lung cancer, breast cancer is also the second leading cause of death from cancer with more than 200,000 new cases and a mortality rate of about 40,000 women per year [1]. According to current projections, approximately one in eight women (12.3%) will be diagnosed with breast cancer at some stage of their lives. To address this problem, a myriad of epigenetic studies have been conducted.

Epigenetics has gained considerable interest in biomedical science as well as cancer prevention and therapy [2]. It refers to the study of heritable changes, such as DNA methylation and histone modifications, in phenotype without altering the DNA sequence. These epigenetic changes have been known to play important roles in the development of different types of cancer, including breast cancers [3]. A number of dietary phytochemicals that are derived from plants have been shown to modulate these epigenetic changes including proanthocyanidins and resveratrol.

Proanthocyanidins refer to a large class of polyphenols called flavanols. Proanthocyanidins can be found in many plants, like apples, cinnamon, aronia fruit, and cocoa beans, but the powerful compound is most abundant in the bark of the maritime pine and in grapes with roughly 60–70% of them being held in the seeds. Grape seed proanthocyanidins (GSPs) are mostly composed of dimers, trimers, tetramers, and oligomers of monomeric catechins and epicatechins [4]. Studies have shown that GSPs are potent antioxidants with many biological properties [5,6,7]. Chief among them are their anticancer effects, which have also been reported in various types of cancer, such as skin cancer and lung cancer, as well as breast cancer [1,8,9,10,11,12].

3,5,4’-trihydroxy-trans-stilbene or resveratrol (Res) is a stilbenoid, a polyphenol, as well as a phytoalexin naturally produced by a number of plants, such as grapes, berries, peanuts, and the roots of Japanese knotweed when under attack by pathogens. But, it is most abundant in the skin of red grapes; thus, it is rich in red wine [13,14]. Like other polyphenols, resveratrol exhibits anticancer properties through a number of epigenetic regulations, among which its ability to inhibit histone deacetylases (HDACs) has been well studied [15,16,17,18,19,20]. Resveratrol and its analogues have also been reported to regulate histone phosphorylation in various cancers [21,22].

Both GSPs and Res have exhibited anti-carcinogenic properties in a number of studies as aforementioned. The effect of GSPs and Res in combination on cancer, however, remains elusive. To investigate this effect along with the underlying mechanisms at the molecular level, the estrogen receptor-negative (ER-), progesterone receptor-negative (PR-), and HER2-negative (HER2-) MDA-MB-231 human breast cancer cells and the ER+, PR+, and HER2-MCF-7 human breast cancer cells were selected for this study. Moreover, since both GSPs and Res are abundant in red grapes and in red wine, which are vastly consumed around the world, it is likely that some of the components that are present in the GSPs act synergistically with Res [23,24,25]. We therefore sought to examine the combined effects and mechanisms of these dietary components on breast cancer cells in humans.

## 2. Results

### 2.1. GSPs and Res Synergistically Inhibit Cell Viability and Proliferation in MDA-MB-231 and MCF-7 Human Breast Cancer Cells

**Figure 1:**
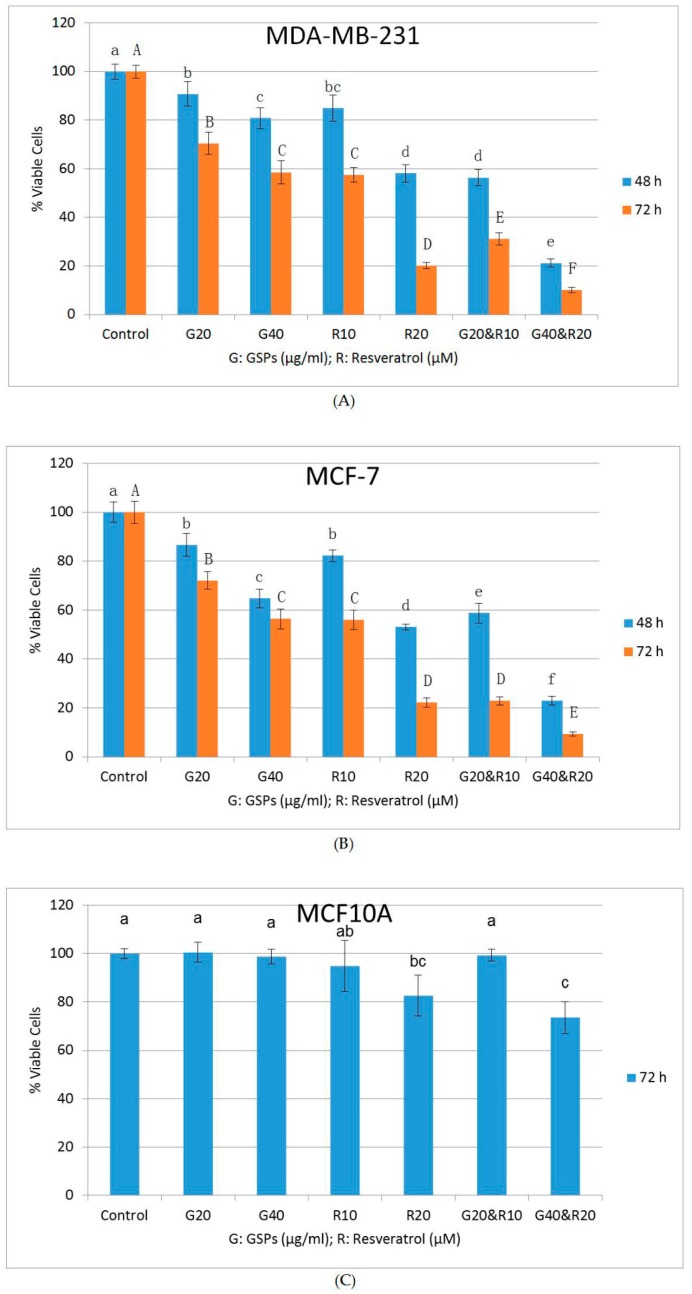
MTT Assay. **I**nhibition of cell viability in MDA-MB-231 (**A**) and MCF-7 (**B**) human breast cancer cells after treatment with grape seed proanthocyanidins (GSPs) (20, 40 μg/ML), Res (10, 20 μM), and their combinations (20 μg/ML GSPs with 10 μM Res, 40 μg/ML GSPs with 20 μM Res) as compared with the dimethyl sulfoxide (DMSO)-treated control cells for 48 h and 72 h. MCF10A human mammary epithelial cells (**C**) were used as the control cells to determine the toxicity of these phytochemicals of varying concentrations. Results were generalized from three independent experiments with very similar observations. The cell viability of each treatment group is represented in percentage compared with the control group as the mean ± SD. Mean values without any same superscript letter (lowercase letters for 48 h in MDA-MB-231 and MCF-7 cells and 72 h in MCF10A cells; uppercase letters for 72 h in MDA-MB-231 and MCF-7 cells) were considered to be significantly different (*p* < 0.05).

To determine the anti-carcinogenic effect of GSPs, Res, and their combinations on human breast cancer cells, an MTT assay was firstly performed. As shown in Figure 1A,B, all treatments with GSPs (20, 40 μg/ML), Res (10, 20 μM), and their combinations (20 μg/ML GSPs with 10 μM Res, and 40 μg/ML GSPs with 20 μM Res) resulted in reduction in cell viability in a dose-and time-dependent manner when compared with the DMSO-treated control groups in MDA-MB-231 and MCF-7 cells. The treatments with GSPs resulted in significant decreases in cell viability by 9% to 19% (*p* < 0.05) after 48 h and 30% to 41% (*p* < 0.05) after 72 h in MDA-MB-231 cells, 13% to 35% (*p* < 0.05) after 48 h and 28% to 44% (*p* < 0.05) after 72 h in MCF-7 cells. The treatments with Res led to significant decreases in cell viability by 15% to 42% (*p* < 0.05) after 48 h and 42% to 80% (*p* < 0.05) after 72 h in MDA-MB-231 cells, 18% to 47% (*p* < 0.05) after 48 h and 44% to 78% (*p* < 0.05) after 72 h in MCF-7 cells. The treatments with GSPs and Res in combinations resulted in a significant decrease in cell viability by 44% to 79% (*p* < 0.05) after 48 h and 69% to 90% (*p* < 0.05) after 72 h in MDA-MB-231 cells, 41% to 77% (*p* < 0.05) after 48 h and 77% to 91% (*p* < 0.05) after 72 h in MCF-7 cells. Furthermore, each combinational treatment exhibited a more significant (*p* < 0.05) reduction in cell viability than treatment with either GSPs or Res alone in both cell lines, suggesting that GSPs and Res inhibited MDA-MB-231 and MCF-7 cells synergistically.

To confirm the synergistic effect on human breast cancer cells between GPSs and SFN, the results from the aforementioned MTT assay were further analyzed by the software CompuSyn version 1.0 (http://www.combosyn.com/) (accessed on 12 October 2014). Combination index (*CI*) values were generated by the software. *CI* < 1 indicates synergism, *CI* = 1 indicates additive effect, *CI* > 1 indicates antagonism [26,27]. As shown in Table 1, all *CI* values of the combinational treatments of the MTT assay exhibited synergism (*CI* > 1) in both MDA-MB-231 and MCF-7 cells.

To investigate the toxicity of GSPs, Res, and their combinations, an MTT assay was performed on the immortalized non-cancerous MCF10A human mammary epithelial cells. The cells were treated with 0.5% (*v/v*) DMSO, GSPs (20, 40 μg/ML), Res (10, 20 μM), and their combinations (20 μg/ML GSPs with 10 μM Res, and 40 μg/ML GSPs with 20 μM Res) for 72 h. As shown in Figure 1C, the lower dose treatments with GSPs (20 μg/ML), Res (10 μM), and their combination exhibited little to no apparent reduction in cell viability when compared with the DMSO-treated control group in MCF10A cells. The higher dose treatments with Res (20 μM) and the combination (40 μg/ML GSPs with 20 μM Res) led to significant decreases in cell viability by 17% and 26% (*p* < 0.05) respectively.

### 2.2. GSPs and Res Synergistically Inhibit Posttreatment Colony Forming Ability in MDA-MB-231 and MCF-7 Human Breast Cancer Cells

**Figure 2:**
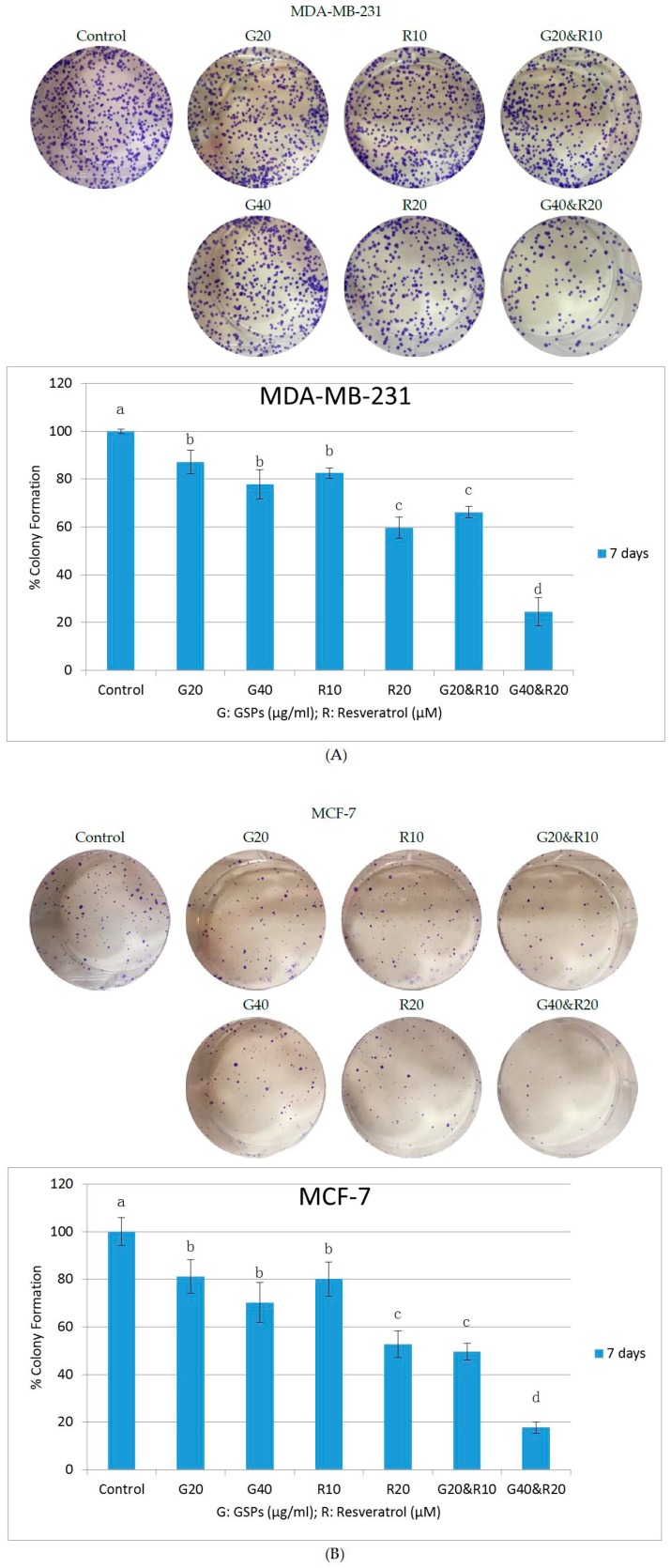

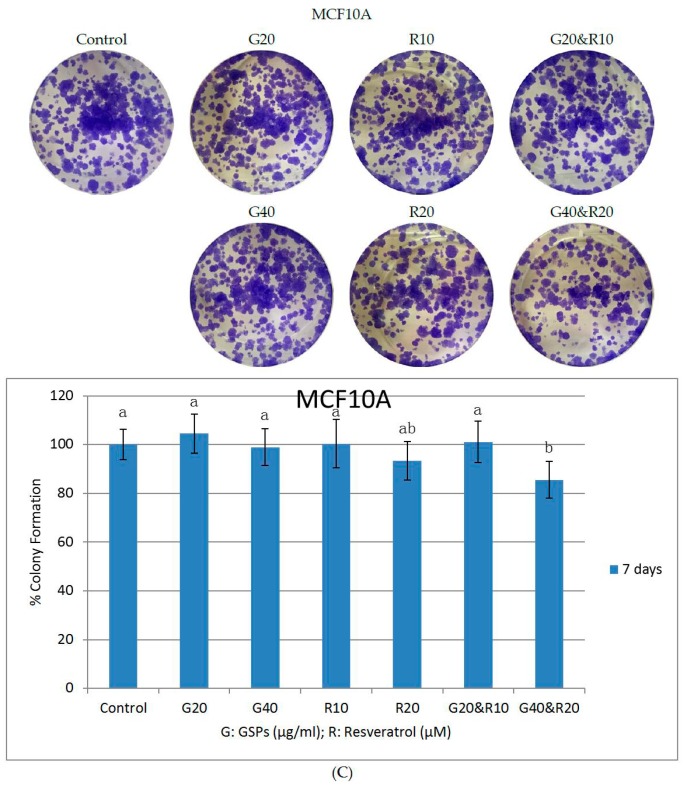
Clonogenic Assay. Inhibition of colony forming ability in MDA-MB-231 (**A**) and MCF-7 (**B**) human breast cancer cells as well as MCF10A (**C**) human mammary epithelial cells in seven days after treatment with GSPs (20, 40 μg/ML), Res (10, 20 μM), and their combinations (20 μg/ML GSPs with 10 μM Res, 40 μg/ML GSPs with 20 μM Res) as compared with the DMSO-treated control groups for 48 h. Results were generalized and representative images were selected from three independent experiments with very similar observations. The colony forming ability of each treatment group is represented in percentage compared with the control group as the mean ± SD. Mean values without any same superscript letter were considered significantly different (*p* < 0.05).

To examine the long-term anti-carcinogenic effect of GSPs, Res, and their combinations on cell proliferation in MDA-MB-231 and MCF-7 human breast cancer cells, clonogenic assays were performed. As indicated in Figure 2, GSPs (20, 40 μg/ML) and Res (10, 20 μM) inhibited the posttreatment colony forming abilities of MDA-MB-231 (A) and MCF-7 (B) cells in a synergistic manner during a seven-day period when compared with the DMSO-treated control groups after treatment for 48 h. The groups previously treated with GSPs showed significant decreases in colony formation by 13% to 22% (*p* < 0.05) in MDA-MB-231 cells and 19% to 30% (*p* < 0.05) in MCF-7 cells. The groups that were formerly treated with Res exhibited significant decreases in colony formation by 17% to 40% (*p* < 0.05) in MDA-MB-231 cells and 20% to 47% (*p* < 0.05) in MCF-7 cells. The pretreatments with GSPs and Res in combinations led to significant reductions in colony formation by 34% to 75% (*p* < 0.05) in MDA-MB-231 cells and 50% to 82% (*p* < 0.05) in MCF-7 cells.

The posttreatment colony forming ability of MCF10A cells was also accessed while using the same method. As shown in Figure 2C, treatments with 20 μg/ML GSPs, 10 μM Res, and their combination expressed no reduction of colony formation. Treatment with 20 μM Res resulted in a 7% reduction (not significant) and the combinational treatment with 40 μg/ML GSPs and 20 μM Res resulted in a 15% reduction (significant, *p* < 0.05) of colony formation in MCF10A cells. Thus, together with the results of the MTT assay, it is safe to conclude that GSPs, Res, and their combinations exhibited no toxicity in lower doses, which had been chosen for the rest of the experiments in this study. As a consequence, the rest of the study proceeded without the use of MCF10A as control cells.

### 2.3. GSPs and Res Synergistically Induce Apoptosis in MDA-MB-231 Human Breast Cancer Cells, Whereas GSPs and Their Combination with Res Inhibit Apoptosis in MCF-7 Human Breast Cancer Cells

**Figure 3:**
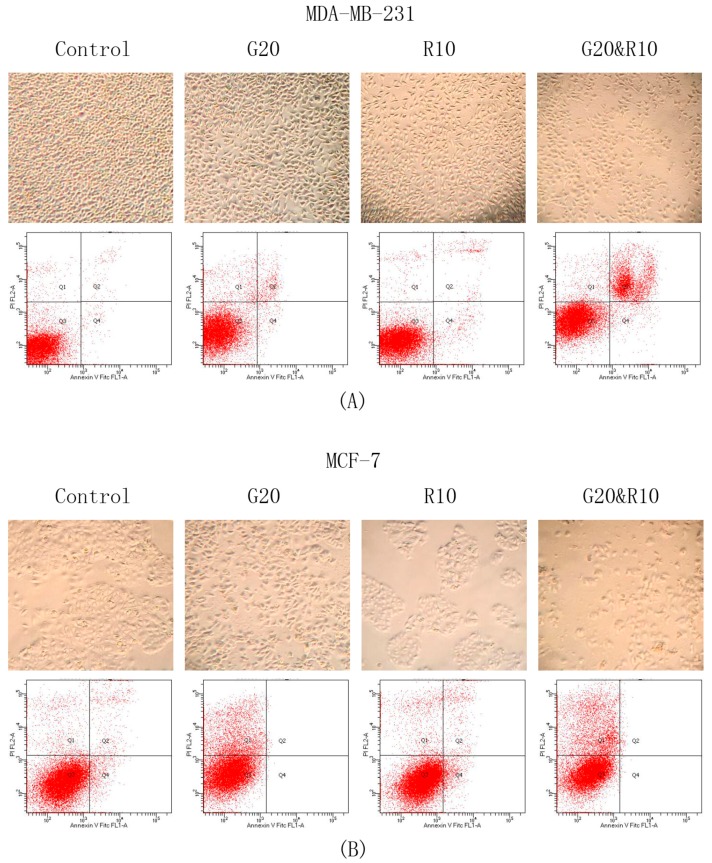

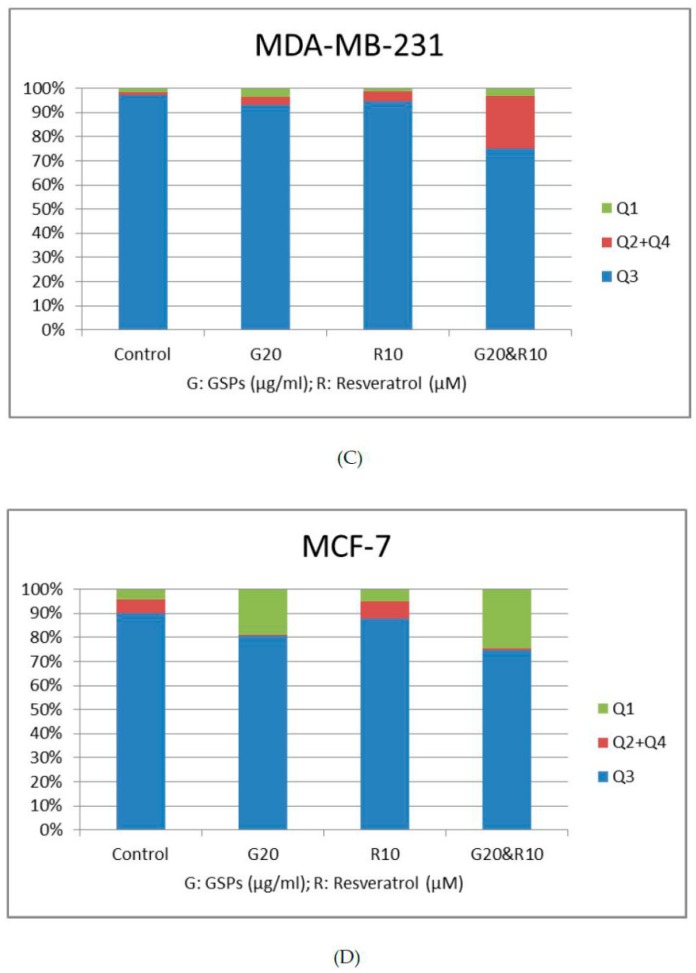
Apoptosis Assay. Morphological and apoptotic changes of MDA-MB-231 (**A**,**C**) and MCF-7 (**B**,**D**) human breast cancer cells induced by GSPs (20 μg/ML), Res (10 μM), and their combination when compared with the DMSO-treated control groups after treatment for 48 h. The images were taken at 40× magnification under a microscope after the cells were treated for 48 h. Apoptosis analysis was performed by the Annexin V-conjugated Alexafluor 488 (Alexa488) Apoptosis Vybrant Assay Kit and analyzed by FACS. Q1 (the upper left quadrant) of the FACS histogram represents dead cells (stained by propidium iodide) that are not associated with apoptosis. Q2 (the upper right quadrant) represents late apoptotic cells (stained by Alexa488 and propidium iodide). Q3 (the lower left quadrant) represents live cells. Q4 (the lower right quadrant) represents early apoptotic cells (stained by Alexa488). The percentage of all four quadrants of cells from each treatment was indicated in C and D. Results were generalized and representative images were selected from three independent experiments with very similar observations.

To investigate whether or not the synergistic effects of GSPs and Res on the inhibition of cell viability and proliferation, as well as on the reduction of posttreatment colony forming ability in MDA-MB-231 and MCF-7 human breast cancer cells are associated with the induction of apoptosis, apoptosis analysis was performed by using the Annexin V-conjugated Alexafluor 488 (Alexa488) Apoptosis Vybrant Assay Kit, following the manufacture’s protocol. Firstly, cell density was reduced in groups treated with GSPs (20 μg/ML) and Res (10 μM) alone and was greatly reduced in the group that was treated with their combination when compared with the DMSO treated control group after treatment for 48 h in both MDA-MB-231 and MCF-7 cells, as shown in Figure 3A,B. Morphological changes were also observed in the phytochemical-treated groups as compared with the control group. Secondly, apoptosis was analyzed using flow cytometry as described above. Cells were counted in four quadrants in the FACS histograms where Q1 (the upper left quadrant) represents dead cells (stained by propidium iodide) that are not associated with apoptosis, Q2 (the upper right quadrant) represents late apoptotic cells (stained by Alexa488 and propidium iodide), Q3 (the lower left quadrant) represents live cells, and Q4 (the lower right quadrant) represents early apoptotic cells (stained by Alexa488). Q2 and Q4 were grouped together when the percentage of all four quadrants of cells from each treatment was illustrated in Figure 3C,D. The results show that the combinational treatment of GSPs and Res significantly induced apoptosis by 21.8% (*p* < 0.05) compared to 3.4% and 4.1% induced by treatment with GSPs and Res alone respectively in MDA-MB-231 cells (Figure 3A,C). However, such induction of apoptosis was not observed in MCF-7 cells as suggested by Figure 3B,D, GSPs, and their combination with Res inhibited apoptosis in MCF-7 cells compared with the groups treated with DMSO and Res alone. Furthermore, GSPs and their combination with Res increased cell death, which was not resulting from apoptosis in MCF-7 cells. The combinational treatment led to 24.6% cell death in MCF-7 cells, which is greater than 19.1% as caused by GSPs and 5.1% s caused by Res combined. All of the evidence suggests that the synergism between GSPs and Res discovered in previous experiments may be associated with the induction of apoptosis in MDA-MB-231 cells but not in MCF-7 cells.

### 2.4. GSPs, Res, and Their Combination Upregulate Bax Expression and Down-Regulate Bcl-2 Expression in MDA-MB-231 Human Breast Cancer Cells, Whereas GSPs and Their Combination with Res Down-Regulate Bax Expression in MCF-7 Human Breast Cancer Cells

**Figure 4:**
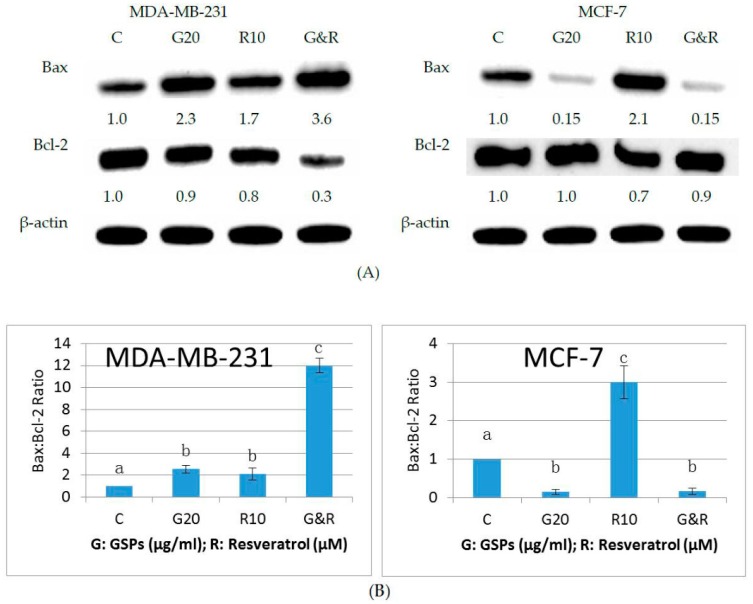
Western Blot Analysis. Change of expression of Bax and Bcl-2 in MDA-MB-231 and MCF-7 human breast cancer cells induced by GSPs (20 μg/ML), Res (10 μM), and their combination when compared with the DMSO-treated control groups after treatment for 48 h (**A**). β-actin was used to confirm equivalent loading of the protein samples. The relative density of each band was measured by ImageJ and was shown under each blot of Bax and Bcl-2 after normalization to the control. A representative image was selected from three independent experiments with very similar results. The Bax:Bcl-2 protein ratio is represented as the mean ± SD (**B**). Mean values without any same superscript letter were considered significantly different (*p* < 0.05).

To verify the results of the apoptosis analysis, western blot analysis was performed to determine the expression of the pro-apoptotic protein Bax and the anti-apoptotic protein Bcl-2 in MDA-MB-231 and MCF-7 human breast cancer cells, as the induction of apoptosis is linked to the upregulation of Bax and to the down-regulation of Bcl-2 [28,29]. As shown in Figure 4A, Res (10 μM) increased Bax expression by 70%, GSPs (20 μg/ML) more than doubled Bax expression, and their combination nearly quadrupled the expression of Bax when compared with the control group treated with DMSO in MDA-MB-231 cells after treatment for 48 h. GSPs and Res decreased Bcl-2 expression by 10% and 20%, respectively, and their combination synergistically decreased Bcl-2 expression by 70% in MDA-MB-231 cells. While, in MCF-7 cells, Bax expression was reduced to 15% in both GSPs treated group and the group treated with GSPs and Res in combination as compared with the control group. Little or no change in the expression of Bcl-2 was detected in the groups that were treated with GSPs either alone or in combination with Res. Although, Res, as in MDA-MB-231 cells, led to a more than two-fold increase in Bax expression and resulted in a 30% decrease in Bcl-2 expression when compared with the control group in MCF-7 cells. Bax:Bcl-2 protein ratio was further calculated in both cell lines, since the ratio is considered to play a determinant role in signal transmission of apoptosis [30]. As displayed in Figure 4B, the Bax:Bcl-2 protein ratio from the combinational treatment group demonstrated a significant increase (*p* < 0.05) when compared to the other groups in MDA-MB-231 cells, while the ratio from the groups that were treated with GSPs either alone or in combination with Res expressed a significant decrease (*p* < 0.05) as compared to the other groups in MCF-7 cells. These results suggest that GSPs and Res synergistically induce apoptosis in MDA-MB-231 cells through promoting transmission of apoptotic signals, whereas GSPs either alone or in combination with Res inhibit apoptosis by suppressing transmission of apoptotic signals in MCF-7 cells.

### 2.5. GSPs, Res, and Their Combination Decrease DNMT Activity as Well as HDAC Activity in MDA-MB-231 and MCF-7 Human Breast Cancer Cells

**Figure 5:**
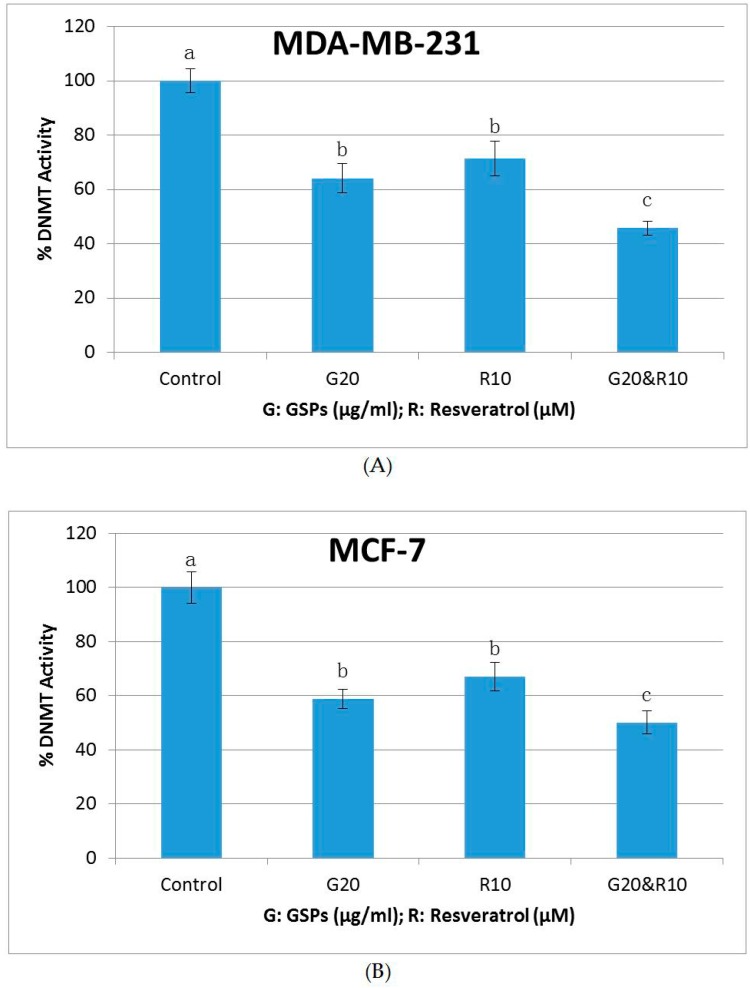
DNA methyltransferases (DNMT) Activity. Inhibition of DNMT activity in MDA-MB-231 (**A**) and MCF-7 (**B**) human breast cancer cells after treatment with GSPs (20 μg/ML), Res (10 μM), and their combination compared with the DMSO-treated control groups for 48 h. Results were generalized from three independent experiments with very similar observations. The DNMT activity of each treatment group is represented in percentage when compared with the control group as the mean ± SD. Mean values without any same superscript letter were considered significantly different (*p* < 0.05).

**Figure 6:**
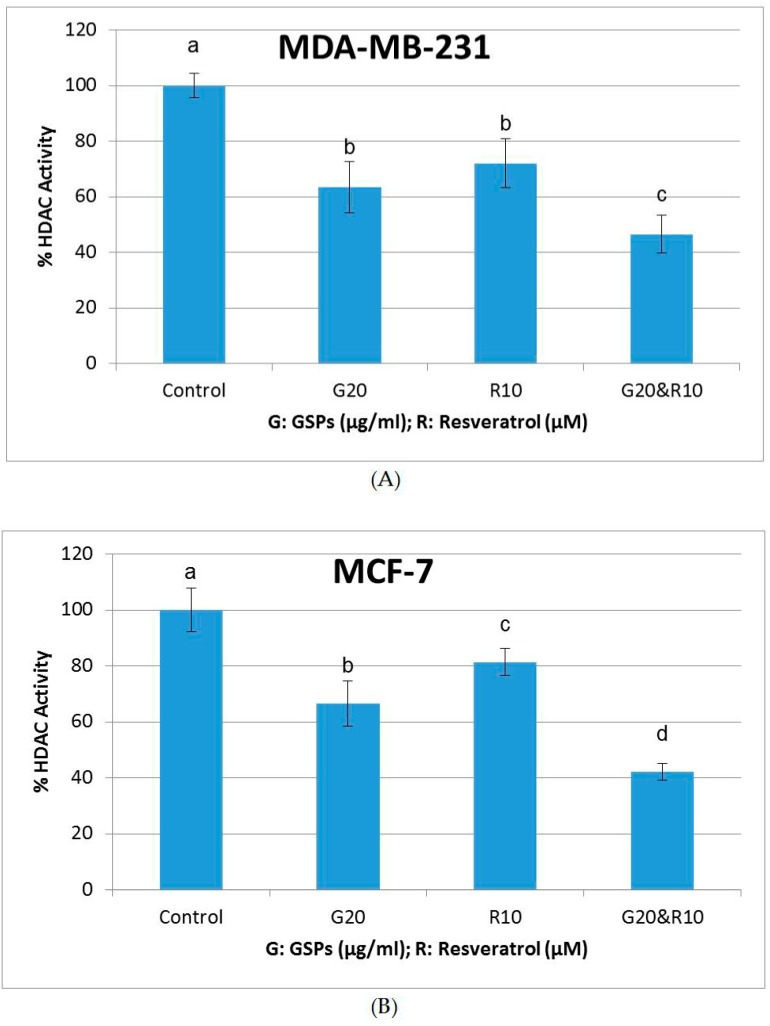
HDAC Activity. Inhibition of HDAC activity in MDA-MB-231 (**A**) and MCF-7 (**B**) human breast cancer cells after treatment with GSPs (20 μg/ML), Res (10 μM), and their combination compared with the DMSO-treated control groups for 48 h. Results were generalized from three independent experiments with very similar observations. The HDAC activity of each treatment group is represented in percentage as compared with the control group as the mean ± SD. Mean values without any same superscript letter were considered significantly different (*p* < 0.05).

To further explore the mechanisms of the inhibitory effects of GSPs and Res on MDA-MB-231 and MCF-7 human breast cancer cells, DNMT and HDAC activity assays were performed. As shown in Figure 5 and Figure 6, GSPs (20 μg/ML), Res (10 μM), and their combination significantly decreased DNMT activity and HDAC activity when compared with the DMSO-treated control group in both MDA-MB-231 and MCF-7 cells after treatment for 48 h (*p* < 0.05). The combinational treatment resulted in greater decreases in DNMT activity and HDAC activity in both cell lines. The inhibitory effect on HDAC activity in MCF-7 cells of the combinational treatment was more than additive (Figure 6B), suggesting an epigenetic mechanism at least for HDACs that could be involved in the effects of these compounds.

## 3. Discussion

In recent years, the effects of dietary components in combination on cancer have gained increasing interest. In this study, we report for the first time the combinational inhibitory effect of grape seed proanthocyanidins (GSPs) and resveratrol (Res) on MDA-MB-231 and MCF-7 human breast cancer cells. We chose GSPs and Res for our study because they are both abundant in grapes, which are some of the most consumed fruits by humans and they are considered to have considerable health benefits. However, most grapes on the market for direct consumption are seedless due to a natural genetic mutation some time ago that prevented the young seeds from maturing and developing a hard coat. Since proanthocyanidins are mostly contained in the seeds of grapes, these seedless grapes, as a result, offer little to no proanthocyanidins. Fortunately, the grapes that are used to produce red wines are seeded and both skin and seeds are preserved and utilized during red wine production. In addition, red wine offers more concentrated GSPs and Res than do red grapes, which makes it more feasible to consume a glass of red wine than a good amount of grapes every day.

Investigations on GSPs and Res have increased during recent years. It has been reported that GSPs dose- and time-dependently inhibited cell viability in human epidermoid carcinoma A431 cells [11]. Our lab previously reported that Res decreased cell viability in HCC1806 and MDA-MB-157 human breast cancer cells in a dose- and time-dependent manner [27]. Such dose- and time-dependent inhibition is also seen in this study in MDA-MB-231 and MCF-7 human breast cancer cells (Figure 1 and Figure 2). However, no study, thus far, has investigated their combinational effects on cancer in humans. It has long been believed that dietary components are easier to absorb and offer better effects in their natural form than in their purified form. One explanation is that there may be other natural compounds that are acting synergistically with the dietary component (s) of interest in their natural form. Our results lend credence to this concept. As shown in our MTT assay (Figure 1), the combinational treatments of GSPs and Res reduced cell viability and proliferation in both MDA-MB-231 and MCF-7 cells significantly more than did treatment with either GSPs or Res of the same concentration alone after 48 h and 72 h. The combination index (*CI*) values generated by the software CompuSyn indicate strong synergism (*CI* < 1) between GSPs and Res (Table 1). Additionally, to determine the long-term effect of GSPs, Res, and their combinations on the posttreatment colony forming ability in MDA-MB-231 and MCF-7 cells, we performed clonogenic assays, in which the cells were treated with the aforementioned phytochemicals at the same concentrations as was used in our MTT assays for 48 h before they were trypsinized, counted and the same number of cells were seeded in fresh media to allow adherence, proliferation, and colony formation for seven days. The results suggest that cell proliferation in MDA-MB-231 and MCF-7 cells was reduced not only under the treatment with GSPs, Res, and their combinations, but after the treatment as well (Figure 2). The dose-dependent inhibition in the MTT assay was also observed in the clonogenic assay, as the groups that were treated with the higher doses of GSPs, Res, and their combination exhibited fewer colonies than these with the lower dose treatments. Also, the posttreatment effect of GSPs and Res on MDA-MB-231 and MCF-7 cells proved to be synergistic. Collectively, the results of the clonogenic assay support our findings in the MTT assay.

We also used the immortalized non-cancerous MCF10A human mammary epithelial control cells to examine the toxicity of the phytochemicals that we used in this study. We report that GSPs at 20 μg/ML, Res at 10 μM, and their combination demonstrated no toxicity in cell viability or posttreatment cell proliferation after 72 h treatment (Figure 1C and Figure 2C). However, Res at 20 μM resulted in a significant decrease in cell viability and a non-significant decrease in posttreatment colony formation when compared to the DMSO-treated control group in MCF10A cells. Moreover, the combination of GSPs at 40 μg/ML and Res at 20 μM rendered significant reductions in cell viability and in posttreatment colony formation. Yet, it may be imprudent to conclude that the combination of GSPs and Res at such concentrations could be toxic since MCF10A cells, though non-carcinogenic, are immortalized. Thus, they are considered to exhibit at least some degree of telomerase activity. We have previously reported that Res (15 μM) down-regulated *hTERT* (telomerase reverse transcriptase in humans) mRNA levels in HCC1806 human breast cancer cells after 72 h treatment [27]. Therefore, it is reasonable to deduce that the presence of Res may have given rise to the inhibition of MCF10A cells through down-regulating *hTERT* expression rather than toxicity. Regardless, we had chosen the lower concentration of GSPs (20 μg/ML), Res (10 μM), and their combination, which led to significant decreases in cell viability and posttreatment colony forming ability in MDA-MB-231 and MCF-7 cells (Figure 1 and Figure 2), for the rest of the experiments in this study.

Both GSPs and Res have been reported to induce apoptosis in human cancer cells [11,27,30]. In this study, we tested their combinational effect on the induction of apoptosis in MDA-MB-231 and MCF-7 human breast cancer cells. Our results show that GSPs (20 μg/ML) and Res (10 μM) synergistically induced apoptosis in MDA-MB-231 cells, but not in MCF-7 cells (Figure 3). Treatment with Res induced apoptosis when compared to the DMSO-treated control group; however, treatments with GSPs alone and in combination with Res almost eliminated both early (Q4) and late (Q2) apoptotic cells in MCF-7 cells. The combination did, however, exhibit a more than additive effect on non-apoptotic cell death (Q1), as shown in Figure 3B,C. We then examined the effects of the phytochemicals on the protein expression of Bax and Bcl-2, since the proteins of the Bcl-2 family are highly associated with the induction of apoptosis. As expected, the results of our Western blot analysis show that GSPs, Res, and their combination upregulated Bax expression and down-regulated Bcl-2 expression in MDA-MB-231 cells (Figure 4A). In addition, the Bax:Bcl-2 protein ratio, which is a determinant role in the signal transmission of apoptosis, was significantly higher after treatment with the combination for 48 h when compared with the groups treated with DMSO, GSPs or Res (Figure 4B), suggesting that GSPs and Res in combination greatly enhance apoptotic signal transmission; thus, apoptosis may contribute to their synergism. In MCF-7 cells, however, GSPs significantly down-regulated Bax expression and caused little to no change in Bcl-2 expression, regardless of the presence of Res in comparison with the DMSO treated control group after 48 h treatment (Figure 4A). The Bax:Bcl-2 protein ratio was significantly lower after being treated with GSPs alone and in combination with Res for 48 h when compared with the DMSO-treated control group (Figure 4B), which indicates that GSPs inhibit apoptosis via blocking apoptotic signal transmission in MCF-7 cells. These findings from the Western blot analysis provide evidence for the results of our apoptosis analysis.

DNA methylation and histone deacetylation have been recognized to be associated with cancer prevention and therapy through regulating the expression of tumor suppressor genes and oncogenes. DNA methyltransferases (DNMTs) and histone deacetylases (HDACs), which are enzymes that play crucial roles in these processes respectively, have been reported to act in collaboration in cancer development [31,32]. Previous studies in our lab have shown that phytochemicals acting as DNMT inhibitors as well as those with HDAC inhibiting properties can work in synergy in inhibiting human cancer [33,34,35]. Thus, we performed DNMT activity assays and HDAC activity assays to further understand the effects of GSPs, Res, and their combination on MDA-MB-231 and MCF-7 human breast cancer cells. The results show that both GSPs and Res acted as strong DNMT inhibitors as well as HDAC inhibitors in MDA-MB-231 and MCF-7 cells, and their combination resulted in a greater reduction in DNMT activity and HDAC activity than did GSPs and Res alone in both of the cell lines (Figure 5 and Figure 6), which may suggest that GSPs and Res synergistically inhibit MDA-MB-231 and MCF-7 cells by upregulating cancer suppressor genes through decreasing DNMT and HDAC activities. These findings help to illuminate understanding of the enzymatic activities of DNMTs and HDACs in these human breast cancer cell lines. Further analysis of the specific epigenetic modifiers in the DNMT family (DNMT1, DNMT3A, and DNMT3B) and HDAC family (HDAC1 etc.) is to be conducted in future studies. It would also be interesting in future studies to investigate which cancer suppressor genes these phytochemicals modulate to give rise to such antagonistic inhibition on these human breast cancer cell lines.

Some studies have shown that MCF-7 human breast cancer cells lack caspase-3 expression and undergo apoptosis through caspase-3-independent pathways [36,37,38]. Another study further investigated whether caspase-3 would affect Bax-induced apoptosis in MCF-7 cells by using normal caspase-3-deficient MCF-7 cells and ones that were transfected with the *CASP3* gene (MCF-7/*CASP3*) [39]. The study showed that the protein expression of Bax was upregulated in both MCF-7 and MCF-7/*CASP3* cells, and that the apoptotic rate was unaffected with caspase-3 expression in MCF-7/*CASP3* cells, suggesting that the lacking caspase-3 did not impact on Bax-induced apoptosis in MCF-7 cells. Therefore, caspase-3 defeciency is unlikely to be a factor contributing to the different apoptotic responses to the treatments with GSPs and their combination with Res between MDA-MB-231 and MCF-7 cell lines.

Another difference worth mentioning between the two cell lines is that MDA-MB-231 cells are estrogen receptor-negative (ER-), whereas MCF-7 cells are ER+. Flavonoids have been reported to play major roles in the reduction of cell proliferation in MCF-7 cells through antiestrogenic activities [40,41,42], and to inhibit 17 β-estradiol-related cancer by reducing ERα signal transduction [43]. Thus, it is reasonable to speculate that GSPs, which mostly consist of flavonoids, may have contributed to the different results from the apoptosis assays in this study between the two cell lines through regulations of estrogenic and/or antiestrogenic activities. The underlying mechanism of these observations is yet to be investigated in future studies. It would also be of great interest to explore the effects of GSPs on the activation of alternative cell death pathways and inhibition of caspase-3-independent apoptotic pathways in the ER+ MCF-7 human breast cancer cells.

## 4. Materials and Methods

### 4.1. Cell Culture and Treatment

The ER-, PR-, and HER2- MDA-MB-231 human breast cancer cells and the ER+, PR+, and HER2- MCF-7 human breast cancer cells were obtained from ATCC (Manassas, VA, USA). The immortalized non-cancerous MCF10A human mammary epithelial cells, which were also obtained from ATCC (Manassas, VA, USA), were used as the control [44]. Both cell lines of human breast cancer were cultured in Dulbecco’s Modified Eagle’s Medium (DMEM) with 10% fetal bovine serum (FBS) and 1% penicillin/streptomycin. The MCF10A control cells were cultured in DMEM/F12 medium with 5% donor horse serum (DHS), 100 ng/ML of cholera endotoxin, 20 ng/ML of epidermal growth factor (EGF), 0.5 μg/ML of hydrocortisone, 2 mM l-glutamine, and 1% penicillin/streptomycin. The penicillin/streptomycin, the DMEM and the DMEM/F12 media were obtained from Mediatech Inc. (Manassas, VA, USA). The FBS and the DHS were obtained from Atlanta Biologicals (Lawrenceville, GA, USA). The rest of the reagents that are listed above were obtained from Sigma-Aldrich (St. Louis, MO, USA). All cells were cultured in an incubator at 37 °C with 5% CO_2_ where humidity was controlled, and sub-cultured at 85–90% confluence. After sub-culturing, all of the cells were given 24 h to adhere and to recover before they were treated with GSPs (20, 40 μg/ML), Res (10, 20 μM), and their combinations (20 µg/ML GSPs with 10 µM Res, 40 µg/ML GSPs with 20 µM Res) for 48 h. Media and treatment agents were refreshed every 24 h for two- and three-day treatments. Dimethyl sulfoxide (DMSO) was used as the vehicle control at the concentration of 0.5% (*v*/*v*) in media.

### 4.2. Chemicals

Grape seed proanthocyanidins (GSPs) (>95% pure) were purchased from Kikkoman Corporation (Tokyo, Japan). Resveratrol (Res) (>99% pure; HPLC) and dimethyl sulfoxide (DMSO) were purchased from Sigma-Aldrich (St. Louis, MO, USA). GSPs were prepared in DMSO and were stored as a stock at the concentration of 100 mg/ML at −20 °C. Resveratrol (Res) was prepared in DMSO and was stored as a stock at the concentration of 100 mM (mmol/L) at −20 °C.

### 4.3. MTT Assay

The number of viable cells in each well was estimated by the uptake of the tetrazolium salt, 3-(4,5-dinethylthiazol-2-yl)-2,5-diphenyltetrazolium bromide (MTT). Approximately 4000 cells per well of each cell line were plated in 96-well plates and incubated for 24 h at 37 °C with 5% CO_2_ to allow for the cells to adhere to the bottom. Then, the cells were treated with 0.5% (*v/v*) DMSO, GSPs (20, 40 μg/ML), Res (10, 20 μM), and their combinations (20 µg/ML GSPs with 10 µM Res, 40 µg/ML GSPs with 20 µM Res) for 48 h and 72 h. After the treatments, the cells were incubated with 100 μL of 1 mg/ML MTT solution for an additional 3.5 h at 37 °C. Thereafter, the MTT solution was aspirated and 150 μL of DMSO was added to each well to dissolve the formazan crystals. Finally, the absorbance was read at 595 nm using the iMark microplate reader (Bio-Rad, Hercules, CA, USA) with the software Microplate Manager 6 (Bio-Rad). Cellular viability was calculated as a percentage relative to the vehicle control treated by DMSO.

### 4.4. Clonogenic Assay

Approximately the same number of cells were treated with 0.5% (*v/v*) DMSO, GSPs (20, 40 μg/ML), Res (10, 20 μM), and their combinations (20 µg/ML GSPs with 10 µM Res, 40 µg/ML GSPs with 20 µM Res) in six-well plates for 48 h at 37 °C with 5% CO_2_. The cells were then harvested and approximately 500 cells of each treatment were seeded in six-well plates with fresh media and were incubated undisturbed at 37 °C with 5% CO_2_ for seven days, during which time the cells were allowed for proliferation and colony formation. Afterwards, the media was aspirated, the colonies were washed with cold phosphate buffer saline, fixed with cold 70% methanol, and were stained with 0.25% trypan blue solution. Finally, photographs were taken and colonies with over 50 cells were counted.

### 4.5. Apoptosis Assay

Apoptosis of breast cancer cells induced by GSPs, Res, and their combinations were quantitatively determined by flow cytometry while using the Annexin V-conjugated Alexafluor 488 (Alexa488) Apoptosis Vybrant Assay Kit (Life Technologies, Carsbald, CA, USA). Approximately 2 × 10^5^ cells were seeded in each well of six-well plates and were left for 24 h at 37 °C with 5% CO_2_ for adherence and recovery. The cells were then treated with 0.5% (*v/v*) DMSO, GSPs (20 μg/ML), Res (10 μM), and their combination for 48 h. Thereafter, the cells were harvested by brief trypsinization, washed with PBS, and incubated at room temperature in the dark for 10 min, during which time the cells were stained with Alexa488 and propidium iodide (PI) in Annexin-binding buffer. The cells were then measured by a fluorescence-activated cell sorting (FACS) machine and analyzed by the CellQuest software version 3.3 from BD Biosciences (San Jose, CA, USA).

### 4.6. Western Blot Analysis

Approximately the same number of cells were treated with 0.5% (*v/v*) DMSO, GSPs (20 μg/ML), Res (10 μM), and their combination in six-well plates for 48 h at 37 °C with 5% CO_2_. The cells were then harvested, and protein extracts were prepared by RIPA lysis buffer (Upstate Biotechnology, Lake Placid, NY, USA), following the manufacturer’s protocol. Protein concentrations were determined by Bradford using the Bio-Rad protein assay (Bio-Rad, Hercules, CA, USA). Protein extract (50 μg) was loaded into a 4–15% Tris-HCl gel (Bio-Rad) and separated by electrophoresis at 200 V until the dye arrived near to the end of the gel. The separated proteins were transferred to a nitrocellulose membrane at 25 V for 10 min by the Trans-Blot Turbo transfer system (Bio-Rad). Afterwards, the membrane was blocked in Tris-buffered saline (TBS) solution with 0.5% dry milk and 0.5% Tween (TBST), following the SNAP i.d. 2.0 protein detection system protocol (EMD Millipore, Billerica, MA, USA). Primary and secondary antibody incubations were performed according to the manufacturer’s protocol. Immunoreactive bands were visualized using Clarity Western ECL Substrate (Bio-Rad).

### 4.7. DNMT Activity Assay

Cells were treated with 0.5% (*v*/*v*) DMSO, GSPs (20 μg/ML), Res (10 μM), and their combination in six-well plates for 48 h at 37 °C with 5% CO_2_. The cells were then harvested, and nuclear extracts were prepared using EpiQuik Nuclear Extraction Kit (EpiGentek, Farmingdale, NY, USA), following the manufacturer’s protocol. DNMT activity assay was performed while using EpiQuik DNA Methyltransferase Activity/Inhibition Colorimetric Assay Kit (EpiGentek), following the manufacturer’s protocol.

### 4.8. HDAC Activity Assay

Cells were treated with 0.5% (*v*/*v*) DMSO, GSPs (20 μg/ML), Res (10 μM), and their combination in six-well plates for 48 h at 37 °C with 5% CO_2_. The cells were then harvested, and nuclear extracts were prepared using EpiQuik Nuclear Extraction Kit (EpiGentek, Farmingdale, NY, USA), following the manufacturer’s protocol. HDAC activity assay was performed while using EpiQuik HDAC Activity/Inhibition Colorimetric Assay Kit (EpiGentek), according to the manufacturer’s protocol.

### 4.9. CompuSyn Analysis

The CompuSyn software version 1.0 (http://www.combosyn.com/) (accessed on 12 October 2014) was used to determine synergism/antagonism of combinational treatments. Combination index (*CI*) values were generated by the software. *CI* < 1 indicates synergism, *CI* = 1 indicates additive effect, *CI* > 1 indicates antagonism [26,27].

### 4.10. Statistical Analysis

All of the results were generalized from at least three independent experiments with very similar observations. Error bars indicate standard deviation. Significance was calculated by one-way ANOVA and post-hoc analysis (Tukey’s HSD test). A *p* value < 0.05 was considered statistically significant.

## Figures and Tables

**Table 1:** Synergism between GSPs and resveratrol (Res) indicated by combination index (*CI*) values.

Cell Line	Treatment Time (h)	Dose GSPs (μg/ML)	Dose Res (μM)	Normalized Effect	*CI* Value
MDA-MB-231	48	20	10	0.56362	0.67285
MDA-MB-231	48	40	20	0.21186	0.54965
MDA-MB-231	72	20	10	0.31165	0.74780
MDA-MB-231	72	40	20	0.10106	0.75330
MCF-7	48	20	10	0.58774	0.99477
MCF-7	48	40	20	0.22958	0.88512
MCF-7	72	20	10	0.22885	0.62425
MCF-7	72	40	20	0.09354	0.70506

The *CI* values were generated by the CompuSyn software from calculating the normalized effect (the effect of treatment with phytochemicals compared with that of treatment with DMSO) of the combinational treatments compared with the normalized effect of the treatments with GSPs and Res alone (not shown in this table) from the data of the MTT assays. *CI* < 1 indicates synergism. *CI* = 1 indicates additive effect. *CI* > 1 indicates antagonism.

## Abbreviations

CI	Combination index
DMSO	Dimethyl sulfoxide
DNMT	DNA methyltransferase
ER	Estrogen receptor
GSPs	Grape seed proanthocyanidins
HDAC	Histone deacetylase
PR	Progesterone receptor
Res	Resveratrol

## References

[1] King M., Chatelain K., Farris D., Jensen D., Pickup J., Swapp A., O’Malley S., Kingsley K. (2007). Oral squamous cell carcinoma proliferative phenotype is modulated by proanthocyanidins: A potential prevention and treatment alternative for oral cancer. BMC Complement. Altern. Med..

[2] Bernstein B.E., Meissner A., Lander E.S. (2007). The mammalian epigenome. Cell.

[3] Egger G., Liang G., Aparicio A., Jones P.A. (2004). Epigenetics in human disease and prospects for epigenetic therapy. Nature.

[4] Ricardo-da-Silva J.M., Rigaud J., Cheynier V., Cheminat A., Moutounet M. (1991). Procyanidin dimers and trimers from grape seeds. Phytochemistry.

[5] Xu Y., Li S., Chen R., Li G., Barish P.A., You W., Chen L., Lin M., Ku B., Pan J. (2010). Antidepressant-like effect of low molecular proanthocyanidin in mice: Involvement of monoaminergic system. Pharmacol. Biochem. Behav..

[6] Aguiar T.R., Vidal C.M., Phansalkar R.S., Todorova I., Napolitano J.G., McAlpine J.B., Chen S.N., Pauli G.F., Bedran-Russo A.K. (2014). Dentin biomodification potential depends on polyphenol source. J. Dent. Res..

[7] Corder R., Mullen W., Khan N.Q., Marks S.C., Wood E.G., Carrier M.J., Crozier A. (2006). Oenology: Red wine procyanidins and vascular health. Nature.

[8] Meeran S.M., Vaid M., Punathil T., Katiyar S.K. (2009). Dietary grape seed proanthocyanidins inhibit 12-O-tetradecanoyl phorbol-13-acetate-caused skin tumor promotion in 7,12-dimethylbenz[a]anthracene-initiated mouse skin, which is associated with the inhibition of inflammatory responses. Carcinogenesis.

[9] Prasad R., Vaid M., Katiyar S.K. (2012). Grape proanthocyanidin inhibit pancreatic cancer cell growth in vitro and in vivo through induction of apoptosis and by targeting the PI3K/Akt pathway. PLoS ONE.

[10] Akhtar S., Meeran S.M., Katiyar N., Katiyar S.K. (2009). Grape seed proanthocyanidins inhibit the growth of human non-small cell lung cancer xenografts by targeting insulin-like growth factor binding protein-3, tumor cell proliferation, and angiogenic factors. Clin. Cancer Res..

[11] Meeran S.M., Katiyar S.K. (2007). Grape seed proanthocyanidins promote apoptosis in human epidermoid carcinoma A431 cells through alterations in Cdki-Cdk-cyclin cascade, and caspase-3 activation via loss of mitochondrial membrane potential. Exp. Dermatol..

[12] Engelbrecht A.M., Mattheyse M., Ellis B., Loos B., Thomas M., Smith R., Peters S., Smith C., Myburgh K. (2007). Proanthocyanidin from grape seeds inactivates the PI3-kinase/PKB pathway and induces apoptosis in a colon cancer cell line. Cancer Lett..

[13] Gu X., Creasy L., Kester A., Zeece M. (1999). Capillary electrophoretic determination of resveratrol in wines. J. Agric. Food Chem..

[14] Baur J.A., Sinclair D.A. (2006). Therapeutic potential of resveratrol: The in vivo evidence. Nat. Rev. Drug Discov..

[15] Li G., Rivas P., Bedolla R., Thapa D., Reddick R.L., Ghosh R., Kumar A.P. (2013). Dietary resveratrol prevents development of high-grade prostatic intraepithelial neoplastic lesions: Involvement of SIRT1/S6K axis. Cancer Prev. Res..

[16] Bourguignon L.Y., Xia W., Wong G. (2009). Hyaluronan-mediated CD44 interaction with p300 and SIRT1 regulates beta-catenin signaling and NFkappaB-specific transcription activity leading to MDR1 and Bcl-xL gene expression and chemoresistance in breast tumor cells. J. Biol. Chem..

[17] Wang R.H., Sengupta K., Li C., Kim H.S., Cao L., Xiao C., Kim S., Xu X., Zheng Y., Chilton B. (2008). Impaired DNA damage response, genome instability, and tumorigenesis in SIRT1 mutant mice. Cancer Cell.

[18] Kim J.E., Kim H.S., Shin Y.J., Lee C.S., Won C., Lee S.A., Lee J.W., Kim Y., Kang J.S., Ye S.K. (2008). LYR71, a derivative of trimeric resveratrol, inhibits tumorigenesis by blocking STAT3-mediated matrix metalloproteinase 9 expression. Exp. Mol. Med..

[19] Venturelli S., Berger A., Bocker A., Busch C., Weiland T., Noor S., Leischner C., Schleicher S., Mayer M., Weiss T.S. (2013). Resveratrol as a pan-HDAC inhibitor alters the acetylation status of histone [corrected] proteins in human-derived hepatoblastoma cells. PLoS ONE.

[20] Scuto A., Kirschbaum M., Buettner R., Kujawski M., Cermak J.M., Atadja P., Jove R. (2013). SIRT1 activation enhances HDAC inhibition-mediated upregulation of GADD45G by repressing the binding of NF-kappaB/STAT3 complex to its promoter in malignant lymphoid cells. Cell Death Dis..

[21] Hong Y.B., Kang H.J., Kim H.J., Rosen E.M., Dakshanamurthy S., Rondanin R., Baruchello R., Grisolia G., Daniele S., Bae I. (2009). Inhibition of cell proliferation by a resveratrol analog in human pancreatic and breast cancer cells. Exp. Mol. Med..

[22] Podhorecka M., Halicka D., Klimek P., Kowal M., Chocholska S., Dmoszynska A. (2011). Resveratrol increases rate of apoptosis caused by purine analogues in malignant lymphocytes of chronic lymphocytic leukemia. Ann. Hematol..

[23] Cerpa-Calderon F.K., Kennedy J.A. (2008). Berry integrity and extraction of skin and seed proanthocyanidins during red wine fermentation. J. Agric. Food Chem..

[24] Fujimaki T., Mori S., Horikawa M., Fukui Y. (2018). Isolation of proanthocyanidins from red wine, and their inhibitory effects on melanin synthesis in vitro. Food Chem..

[25] Sun B., de Sa M., Leandro C., Caldeira I., Duarte F.L., Spranger I. (2013). Reactivity of polymeric proanthocyanidins toward salivary proteins and their contribution to young red wine astringency. J. Agric. Food Chem..

[26] Chou T.C. (2008). Preclinical versus clinical drug combination studies. Leuk. Lymphoma.

[27] Kala R., Shah H.N., Martin S.L., Tollefsbol T.O. (2015). Epigenetic-based combinatorial resveratrol and pterostilbene alters DNA damage response by affecting SIRT1 and DNMT enzyme expression, including SIRT1-dependent gamma-H2AX and telomerase regulation in triple-negative breast cancer. BMC Cancer..

[28] Oltvai Z.N., Milliman C.L., Korsmeyer S.J. (1993). Bcl-2 heterodimerizes in vivo with a conserved homolog, Bax, that accelerates programmed cell death. Cell.

[29] Zhan Q., Fan S., Bae I., Guillouf C., Liebermann D.A., O’Connor P.M., Fornace A.J. (1994). Induction of bax by genotoxic stress in human cells correlates with normal p53 status and apoptosis. Oncogene.

[30] Mantena S.K., Baliga M.S., Katiyar S.K. (2006). Grape seed proanthocyanidins induce apoptosis and inhibit metastasis of highly metastatic breast carcinoma cells. Carcinogenesis.

[31] Chen H., Landen C.N., Li Y., Alvarez R.D., Tollefsbol T.O. (2013). Enhancement of Cisplatin-Mediated Apoptosis in Ovarian Cancer Cells through Potentiating G2/M Arrest and p21 Upregulation by Combinatorial Epigallocatechin Gallate and Sulforaphane. J. Oncol..

[32] Xu S., Ren J., Chen H.B., Wang Y., Liu Q., Zhang R., Jiang S.W., Li J. (2014). Cytostatic and apoptotic effects of DNMT and HDAC inhibitors in endometrial cancer cells. Curr. Pharm Des..

[33] Li Y., Meeran S.M., Tollefsbol T.O. (2017). Combinatorial bioactive botanicals re-sensitize tamoxifen treatment in ER-negative breast cancer via epigenetic reactivation of ERalpha expression. Sci. Rep..

[34] Li Y., Buckhaults P., Cui X., Tollefsbol T.O. (2016). Combinatorial epigenetic mechanisms and efficacy of early breast cancer inhibition by nutritive botanicals. Epigenomics.

[35] Royston K.J., Udayakumar N., Lewis K., Tollefsbol T.O. (2017). A Novel Combination of Withaferin A and Sulforaphane Inhibits Epigenetic Machinery, Cellular Viability and Induces Apoptosis of Breast Cancer Cells. Int. J. Mol. Sci..

[36] Wang S., He M., Li L., Liang Z., Zou Z., Tao A. (2016). Cell-in-Cell Death Is Not. Restricted by Caspase-3 Deficiency in MCF-7 Cells. J. Breast Cancer..

[37] Tewari M., Quan L.T., O'Rourke K., Desnoyers S., Zeng Z., Beidler D.R., Poirier G.G., Salvesen G.S., Dixit V.M. (1995). Yama/CPP32 beta, a mammalian homolog of CED-3, is a CrmA-inhibitable protease that cleaves the death substrate poly(ADP-ribose) polymerase. Cell.

[38] Janicke R.U., Sprengart M.L., Wati M.R., Porter A.G. (1998). Caspase-3 is required for DNA fragmentation and morphological changes associated with apoptosis. J. Biol. Chem..

[39] Kagawa S., Gu J., Honda T., McDonnell T.J., Swisher S.G., Roth J.A., Fang B. (2001). Deficiency of caspase-3 in MCF7 cells blocks Bax-mediated nuclear fragmentation but not cell death. Clin. Cancer Res..

[40] Han D.H., Denison M.S., Tachibana H., Yamada K. (2002). Relationship between estrogen receptor-binding and estrogenic activities of environmental estrogens and suppression by flavonoids. Biosci. Biotechnol. Biochem..

[41] Collins-Burow B.M., Burow M.E., Duong B.N., McLachlan J.A. (2000). Estrogenic and antiestrogenic activities of flavonoid phytochemicals through estrogen receptor binding-dependent and -independent mechanisms. Nutr. Cancer.

[42] So F.V., Guthrie N., Chambers A.F., Carroll K.K. (1997). Inhibition of proliferation of estrogen receptor-positive MCF-7 human breast cancer cells by flavonoids in the presence and absence of excess estrogen. Cancer Lett..

[43] Virgili F., Acconcia F., Ambra R., Rinna A., Totta P., Marino M. (2004). Nutritional flavonoids modulate estrogen receptor alpha signaling. IUBMB Life.

[44] Meeran S.M., Patel S.N., Tollefsbol T.O. (2010). Sulforaphane causes epigenetic repression of hTERT expression in human breast cancer cell lines. PLoS ONE.

